# A chronic non‐healing ulcer plantar

**DOI:** 10.1002/ski2.280

**Published:** 2023-12-27

**Authors:** Luisa Unterluggauer, Ichiro Okamoto

**Affiliations:** ^1^ Department of Dermatology Medical University of Vienna Vienna Austria

## Abstract

Acral melanoma is challenging in two ways: it is in some cases difficult to diagnose and, once metastases have occurred, the prognosis is poor as therapy is less effective compared to melanoma from other parts of the skin. Here we report a case, were the correct diagnosis was made after melanoma has spread already to distant sites. Instead of surgery, we decided to start with immunotherapy consisting of Ipilimumab and Nivolumab. A complete response could be achieved without surgery of any tumors, including the primary melanoma.

## Graphical abstract

A 70‐year‐old woman presented with an ulcerating lesion on her right foot which has been treated as a nonhealing‐ulcer for 3 years. A representative biopsy revealed the diagnosis of an acral stage IV melanoma.

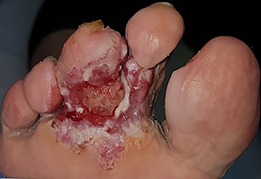

A 70‐year‐old woman presented with an ulcerating lesion on her right foot which has been treated as a nonhealing‐ulcer for 3 years. She was diagnosed with peripheral arterial occlusive disease and recanalisation of the right arteria femoralis superficialis and implantation of a stentgraft was performed. A representative biopsy finally revealed the diagnosis of an acral melanoma with a Breslow depth of 5.04 mm. Staging detected non regional macroscopic lymph node metastases, and S100 was elevated (0.6 μg/L). Due to the size of the primary tumour as well as the distant metastases, an inoperable stage IV melanoma was declared, and a multidisciplinary tumorboard decision for treatment with checkpoint inhibitors Ipilimumab/Nivolumab was made. This left the option open of later surgery after initiation of systemic therapy, similar to a neo‐adjuvant setting and primary amputation/semi‐amputation of the foot could be avoided. Though check point inhibitors show lower effectiveness in acral melanoma compared to melanomas from other part of the skin, after each cycle, the tumour showed a remarkable response to the treatment with regression of the primary tumour as well as lymph node metastases. No residual tumour was detected after 4 cycles of Ipilimumab and Nivolumab. Pictures from before treatment (Figure 1) and after the fourth cycle of Ipilimumab and Nivolumab are shown (Figure 2). The patient remained tumour free until the most recent CT scan 14 months after initiation of therapy and 5 years after evolving of the lesion. This case demonstrates that the use of check point inhibitors even before removal of the primary melanoma can be effective making surgery unnecessary.

**FIGURE 1:**
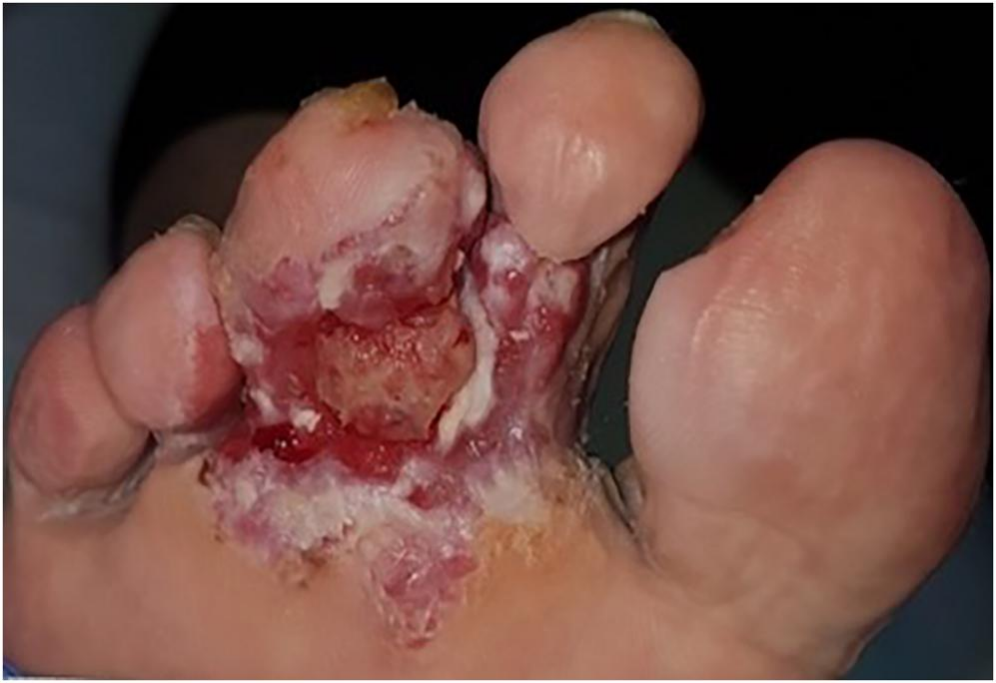
Clinical picture at time of diagnosis.

**FIGURE 2:**
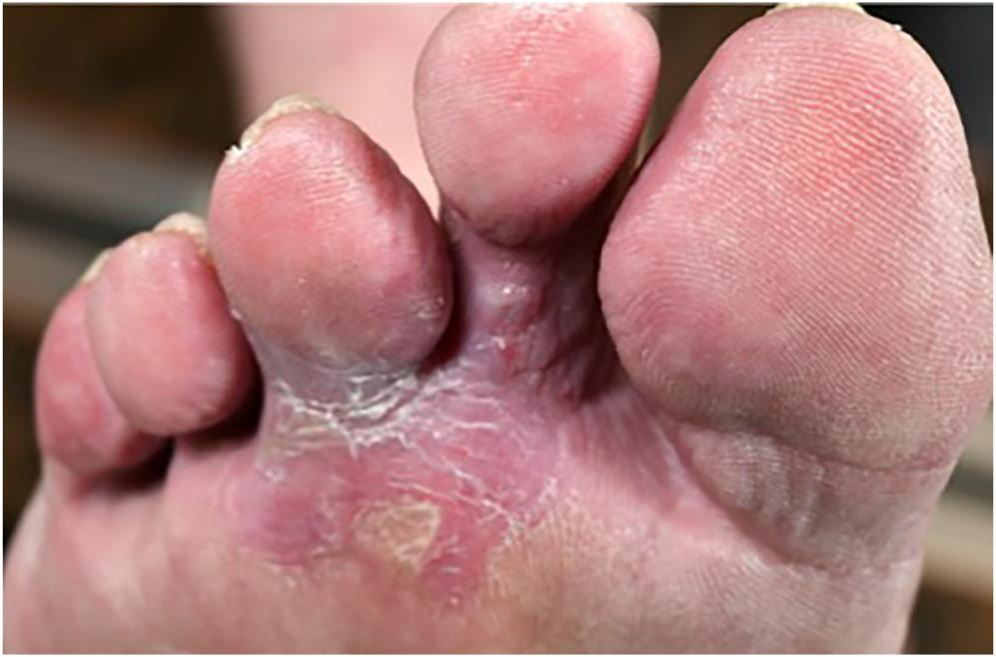
Clinical picture after the fourth cycle of Ipilimumab and Nivolumab.

The lack of improvement after adequate treatment of a non‐healing ulcer should raise the therapists doubts and awareness for a possible malignant aetiology. Biopsy should be taken in the edge area of the ulcerating lesion, to avoid misdiagnosis of an ulcerating lesion.

## AUTHOR CONTRIBUTIONS

**Luisa Unterluggauer**: Writing – original draft (lead). **Ichiro Okamoto**: Writing – review & editing (lead).

## CONFLICT OF INTEREST STATEMENT

None to declare.

## ETHICS STATEMENT

Written and signed consent to publish the information from the patient was obtained.

## Data Availability

No data are available.

